# Design and Locomotion Study of Two-DOF Actuator Driven by Piezoelectric–Electromagnetic Hybrid Mode

**DOI:** 10.3390/s22103739

**Published:** 2022-05-14

**Authors:** Zheng Li, Zhirong Su, Haibo Wang, Shenhui Du, Hexu Sun

**Affiliations:** School of Electrical Engineering, Hebei University of Science and Technology, Shijiazhuang 050018, China; suzhirong@stu.hebust.edu.cn (Z.S.); wanghaibo2021102@163.com (H.W.); dushenhui@hebust.edu.cn (S.D.)

**Keywords:** hybrid drive, two-DOF, piezoelectric, amplification structure, electromagnetic

## Abstract

A piezoelectric actuator (PEA) has the characteristics of high control precision and no electromagnetic interference. To improve the degree of freedom (DOF) to adapt to more working scenes, a piezoelectric–electromagnetic hybrid-driven two-DOF actuator is proposed. The PEA adopts the composite structure of the lever amplification mechanism and triangular amplification mechanism. The structure effectively amplifies the output displacement of the piezoelectric stack and increases the clamping force between the driving foot and the mover. The electromagnetic actuator (EMA) adopts a multi-stage fractional slot concentrated winding permanent magnet synchronous actuator, which can better match the characteristics of PEA. The structure and working principle of the actuator are introduced, the dynamic analysis is carried out, and the factors affecting the clamping force are obtained. At the same time, the air gap magnetic field is analyzed, and the structural size of the actuator is optimized. The experiment shows that the maximum driving speed can reach 348 mm/s, the load capacity is 3 kg, the optimal initial rotor angle is 49°, the maximum torque is 2.9 N·m and the maximum speed is 9 rad/s, which proves the stability and feasibility of the actuator.

## 1. Introduction

PEA has the advantages of fast response speed, strong anti-electromagnetic interference ability, and high control precision [1,2,3]. It is widely used in aerospace, electrical control, biochemistry, medical devices, manufacturing of precision instruments, robots, and other fields [4,5,6].

Since many fields involve the linear motion and rotary motion of actuators, the actuators need to be improved to adapt to more application scenarios [7,8,9]. PEA can be divided into the single-DOF type and multi-DOF type. The multi-DOF type has a compact structure and high torque, but the structure is not flexible, and the control is complex [10,11,12]. Most of them work in resonant mode, which has high requirements on the operating frequency. Therefore, it cannot meet the needs of some scenes. To solve the problem of poor adaptability of multi-DOF actuators, it can be studied based on single-DOF actuators [13,14,15,16]. Although the single-DOF actuators have a single driving method, due to their flexible structure, they can cooperate with other actuators to achieve the purpose of multi-DOF driving.

PEAs work by using the inverse piezoelectric effect of piezoelectric materials [17,18]. This is the phenomenon of material deformation when voltage is applied to piezoelectric materials [19,20]. PEAs utilize the deformation of piezoelectric materials in the driving process to cooperate with other structures and achieve the driving purpose through the friction between the driving foot and the mover [21,22,23]. Because the displacement generated by each friction is very small, the actuator needs to be driven step by step to achieve more obvious output displacement, so the PEAs usually have a low speed. However, it is also because of this characteristic that the PEAs can achieve high-precision output [24,25]. The structure of PEAs is flexible and can realize high-precision control, but it is greatly affected by vibration and their output torque is not high. The EMAs can realize high torque and low speed and can also avoid some effects caused by vibration. Therefore, it is possible to combine the above two actuators and study them.

In this paper, a two-DOF actuator driven by a piezoelectric–electromagnetic hybrid is proposed. Compared with the two-DOF pure EMAs, the displacement resolution of the actuator is improved, and the control accuracy is improved. The starting and braking performance is optimized, so the response speed of the actuator is improved. Compared with the two-DOF pure PEAs [26,27], the actuator can reduce the influence of vibration, and improve the output speed.

The piezoelectric–electromagnetic hybrid actuator consists of the piezoelectric driving part and the electromagnetic driving part. The actuator dimensions of the two driving parts need to be matched for the actuator to have good output performance. The output performance of traditional PEA is low. To solve this problem, a two-stage flexure hinge amplification structure is added to the PEA, which is composed of a lever amplification mechanism and a triangular amplification structure. The structure effectively amplifies the output displacement of the piezoelectric stack and increases the clamping force between the driving foot and the mover. The EMA adopts a multi-stage fractional slot concentrated winding permanent magnet synchronous actuator, which can better match the characteristics of the PEA. Firstly, the structure and working principle of the actuator are introduced, and then the actuator is analyzed and optimized through simulation. Finally, the driving performance of the hybrid actuator is tested by experiments to verify its feasibility.

## 2. Structure and Principle of the Proposed Actuator

**Figure 1:**
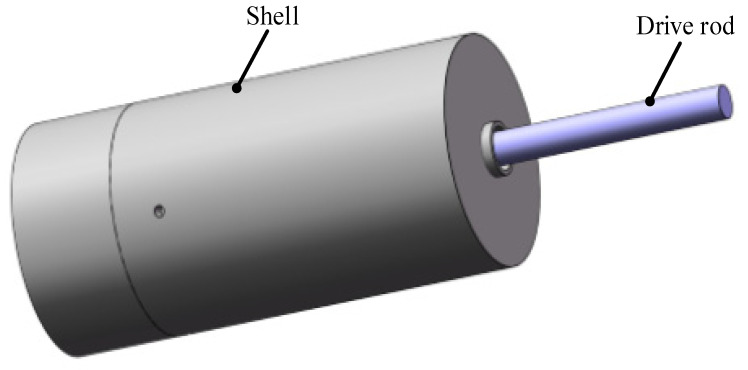
The external structure of the actuator.

Due to there being many parts used in the proposed actuator, some parts need to be fixed, and a nested combination is also required between parts. To facilitate the design requirements, the actuator is designed as a barrel-shaped packaging structure, as shown in Figure 1. The actuator is composed of an electromagnetic part and a piezoelectric part. The electromagnetic part drives the drive rod to rotate, and the piezoelectric part provides the expansion and contraction of the rod to realize the demand of two-DOF drive.

**Figure 2:**
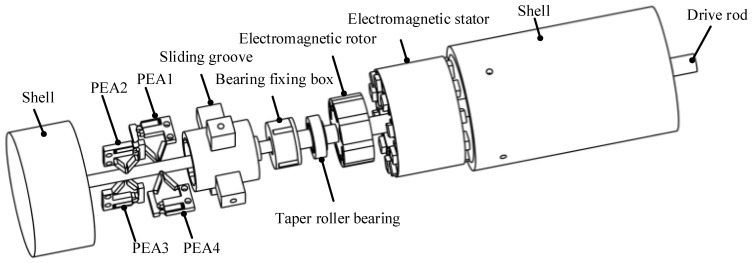
The internal structure of the actuator.

The internal structure of the actuator and the relative position between parts are shown in Figure 2. The parts in the shell from left to right are PEA, sliding groove box, bearing fixing box, tapered roller bearing, electromagnetic rotor, electromagnetic stator, and driving rod. The piezoelectric driving part is composed of four PEAs, PEA1 and PEA4 are a group, which shifts the driving rod to the right during operation; PEA2 and PEA3 are a group, which shifts the driving rod to the left during operation.

**Figure 3:**
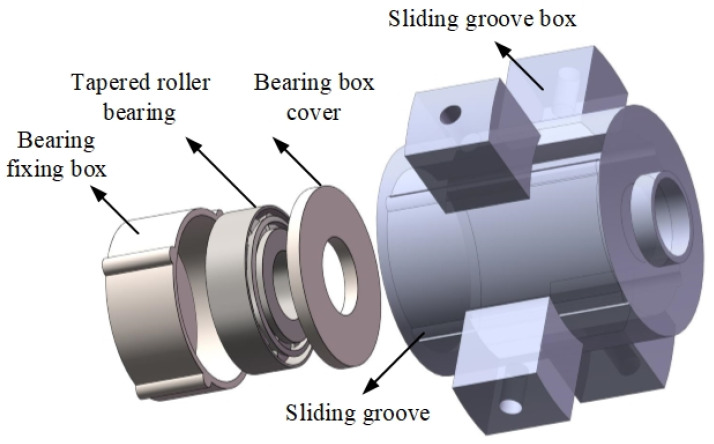
The layout of the bearing devices.

The connection of the piezoelectric part and the electromagnetic part is completed by a set of bearing devices, as shown in Figure 3. The tapered roller bearing is adopted as the bearing, which can be applied to the multi-DOF motion of the drive rod. The bearing fixing box is used to install and fix the bearing, and the external protrusion is used to assemble the groove in the sliding groove box. The inside of the sliding groove box is provided with a groove to provide the up and down sliding motion of the bearing fixing box in the groove, and the outside is provided with four protruding blocks of the support box, which are fixed on the shell by screws.

The EMA is composed of an electromagnetic stator and an electromagnetic rotor, wherein the electromagnetic rotor is connected with the drive rod, and the electromagnetic stator is fixed in the shell. The drive rod is divided into two parts. The left side of the rod of the piezoelectric part is installed in the groove at the bottom of the shell to support and fix the position of the rod, and the other side is connected with the bottom of the bearing box. The left side of the drive rod of the electromagnetic part is bonded with the bearing in the bearing box, and the other side is the embodiment of the final drive of the actuator. The piezoelectric rod initiates telescopic movement along the axial direction and drives the electromagnetic rod to demonstrate the same telescopic movement through contact. At the same time, the electromagnetic rod itself has a rotary movement. Therefore, the actuator can finally drive the output rod to realize the movement of two-DOF.

## 3. Piezoelectric Part

### 3.1. Structure of the PEA

**Figure 4:**
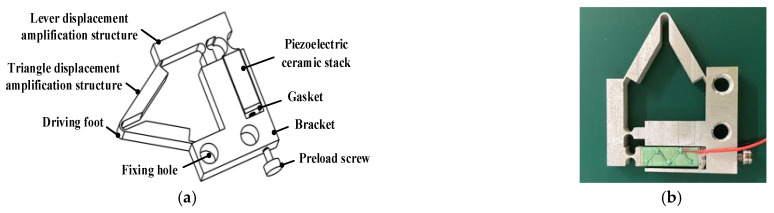
(**a**) Structure diagram of the PEA; (**b**) The prototype of the PEA.

Since the structures of the four PEAs constituting the piezoelectric part are the same, only the structure of one PEA is analyzed. The PEA is composed of a two-stage amplification structure, a piezoelectric ceramic stack, a bracket, a gasket, and a pre-tightening screw, as shown in Figure 4a. The two-stage magnification mechanism is composed of a lever displacement magnification structure and a triangular displacement magnification structure. As the driving element of the PEA, one end of the piezoelectric ceramic stack is bonded with one end of the lever displacement amplification structure using an epoxy resin adhesive. The other end is bonded with the metal gasket to protect the piezoelectric stack when the preloading screw applies preload to the piezoelectric stack. The prototype of the PEA is shown in Figure 4b.

The PEA is composed of a piezoelectric stack and a displacement amplification structure. Since the elongation of the piezoelectric stack itself is not enough to ensure the clamping force with the mover, it needs to be combined with the displacement amplifier. In the method of amplifying the output displacement of a piezoelectric stack, a flexure hinge amplifier has the advantages of high efficiency, no backlash, and is easy to manufacture [28,29]. Different hinged displacement amplifiers are used in different fields according to their shapes. They are composed of simple levers, bridges, and four-bar mechanisms. The lever hinge displacement amplification structure can produce a large output displacement and has high efficiency. Moreover, the triangular displacement amplification structure can also change the direction of amplified displacement. To combine the advantages of the two, a high gain displacement amplification mechanism based on a lever hinge and a triangular hinge is designed for the PEA.

### 3.2. Theoretical Analysis

The piezoelectric stack is composed of a large number of stacked layers, which can be considered a continuous structure.

**Figure 5:**
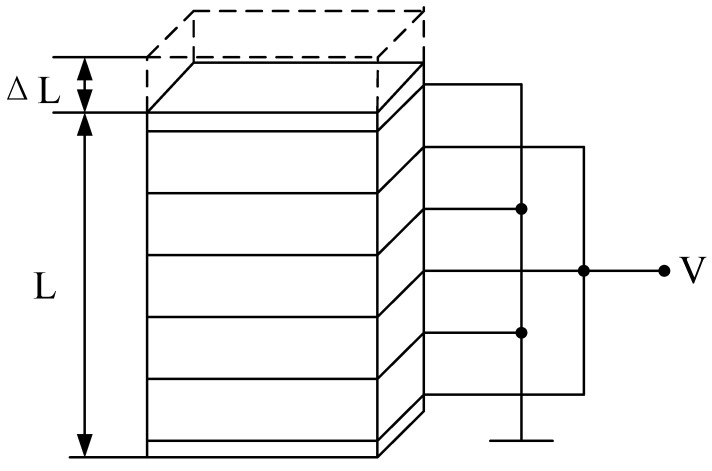
Piezoelectric stack model.

Each layer in the piezoelectric stack alternates between the positive electrode and the zero electrodes, and the layers are mechanically connected in series and electrically connected in parallel. Therefore, significant electron displacement can occur at a relatively low voltage. It is assumed that the PEA is a uniform beam with the cross-sectional area, material density, and Young’s modulus under axial force. In order to show that the material is uniform and isotropic, the mass and elasticity are uniformly distributed throughout the piezoelectric stack. The relationship between stress and strain follows Hooke’s law. A mathematical model is established, as shown in Figure 5.

The piezoelectric ceramic stack produces a longitudinal vibration, and the calculation formula of its longitudinal elongation is as follows [30]:

(1) $$\Delta L=nd_{3,3}U,$$

where Δ*L* represents the longitudinal elongation of the piezoelectric stack, and *L* is the initial length of the piezoelectric stack. *U* is the voltage applied to the piezoelectric stack. *n* is the number of piezoelectric layers, and *d*_3__,__3_ is the piezoelectric constant of the piezoelectric material. Under all strain constraints, the maximum relative displacement is in the range 0.1–0.13%. Therefore, the displacement of a 100 mm long piezoelectric stack is at least 100 μm. Piezoelectric stacks with these parameters can reach about one thousand layers.

**Figure 6:**
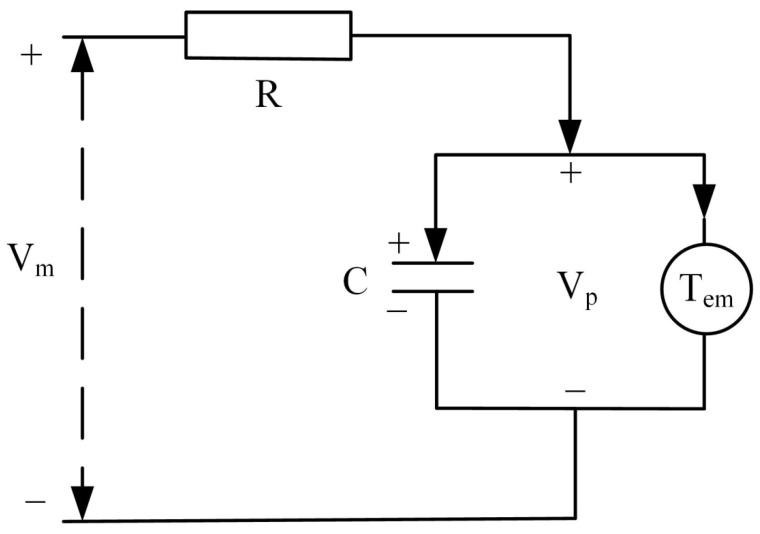
Equivalent circuit of the piezoelectric stack.

The piezoelectric stack operates through the inverse piezoelectric effect of piezoelectric materials. The inverse piezoelectric effect is the deformation caused by mechanical movement by applying a voltage to piezoelectric materials. According to the principle, the operation of the piezoelectric stack is presented in the form of an equivalent circuit, as shown in Figure 6.

The following relationship can be obtained by analyzing the circuit:

(2) $$V_{in}=V_{p}+RC\overset{•}{V_{p}},$$

(3) $$V_{p}T_{em}=F_{PTZ},$$

where *V_in_* is the total voltage provided by the signal generator, and *R* is the equivalent resistance of the signal source, voltage amplifier, and piezoelectric stack. *C* is the equivalent capacitance in the direction of movement of the piezoelectric stack. *V_p_* is the conversion voltage of the piezoelectric stack using the inverse piezoelectric effect. $\overset{•}{V_{p}}$ is the first derivative of *V_p_* with respect to time. *T_em_* is the electromechanical conversion rate based on the piezoelectric effect. *F_ptz_* is the output force of the piezoelectric stack.

### 3.3. Analysis and Simulation of PEA

#### 3.3.1. Dynamic Model Analysis

**Figure 7:**
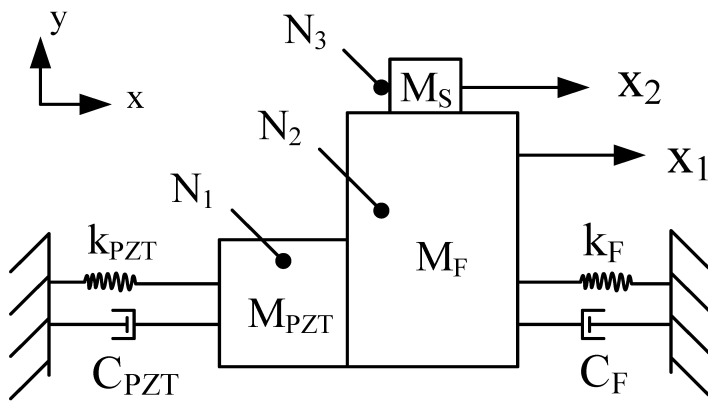
Simplified dynamic model.

In order to be used to study the effect of design parameters on the PEA and provide guidance for future control systems, a simplified dynamic model is established [31,32,33], as shown in Figure 7. According to the simplified dynamic model, the force interaction between the piezoelectric stack N_1_, the support mechanism N_2,_ and the mover N_3_ is generated.

The stress equation is as follows:

(4) $$\begin{array}{c} M_{PZT}\overset{••}{x_{1}}+C_{PZT}{\overset{•}{x}}_{1}+k_{PZT}x_{1}=F_{PZT}-F_{N}, \end{array}$$

(5) $$\begin{array}{ccc} M_{F}{\overset{••}{x}}_{1}+C_{F}\overset{•}{x_{1}}+k_{F}x_{1} & = & F_{N}-f, \end{array}$$

(6) $$\begin{array}{ccc} M_{s}\overset{••}{x_{2}} & = & f, \end{array}$$

In the above formula, *M_PZT_* is the mass of the piezoelectric stack, *k_PZT_* is the stiffness of the piezoelectric stack, and *C_PZT_* is the damping coefficient of the piezoelectric stack. *M_F_* is the mass of the support mechanism, *K_F_* is the stiffness of the support mechanism, and C_F_ is the damping coefficient of the support mechanism. *M_S_* is the mass of the mover. *F_PZT_* is the force generated by the piezoelectric stack. *F_N_* is the force between the piezoelectric stack and the hinge structure, and *f* is the friction between the driving foot and the contact surface of the mover. *X*_1_ is the vector displacement of the hinge structure. *X*_2_ is the vector displacement of the mover. $\overset{•}{x_{1}}$ is the first derivative of the vector displacement *x*_1_ with respect to time. $\overset{••}{x_{1}}$, $\overset{••}{x_{2}}$ is the second derivative of the vector displacement *x*_1,_ *x*_2_ with respect to time.

The friction *f* is derived from the LuGre friction model as follows:

(7) $$\begin{array}{ccc} f & = & \sigma_{0}z+\sigma_{1}\frac{dz}{dt}+\sigma_{2}v, \end{array}$$

(8) $$\begin{array}{ccc} \frac{dz}{dt} & = & v-\frac{\left|v\right|}{g(v)}z, \end{array}$$

(9) $$\begin{array}{ccc} \sigma_{0}g\left(v\right) & = & f_{c}+\left(f_{s}-f_{c}\right)e^{-{\left(v/v_{s}\right)}^{2}}, \end{array}$$

*σ*_0_ is the bristle stiffness, *σ*_1_ is the main damping coefficient, *σ*_2_ is the coefficient of viscous friction, *t* is the time taken for the PEA deformation, *v* is the relative velocity, vs. is the Stribeck velocity, *z* is the main deformation, *f_c_* is the Coulomb friction, *f_s_* is the static friction, and *g*(*v*) is a function based on v.

*x*_2_ is usually much less than *x*_1_, and the value of friction force *f* obtained from the formula is much less than *F_N_* and *F_PZT_*. Therefore, *f* can be omitted in the process of Laplace transformation of the formula. The formula after transformation is as follows:

(10) $$\frac{X_{1}\left(s\right)}{U_{0}\left(s\right)}=\frac{nd_{33}k_{A}}{RCs+1}\times \frac{k_{PZT}}{\left(M_{PZT}+M_{F}\right)s^{2}+\left(C_{PZT}+C_{F}\right)s+\left(k_{PZT}+k_{F}\right)},$$

*R* is the resistance of the equivalent circuit, *C* is the capacitance of the equivalent circuit, and *s* is a variable in the complex frequency domain. *U*_0_ is the voltage value provided by the signal generator to the piezoelectric stack. *k_A_* is the amplification factor of the voltage amplifier.

#### 3.3.2. Kinematic Analysisof the PEA

**Figure 8:**
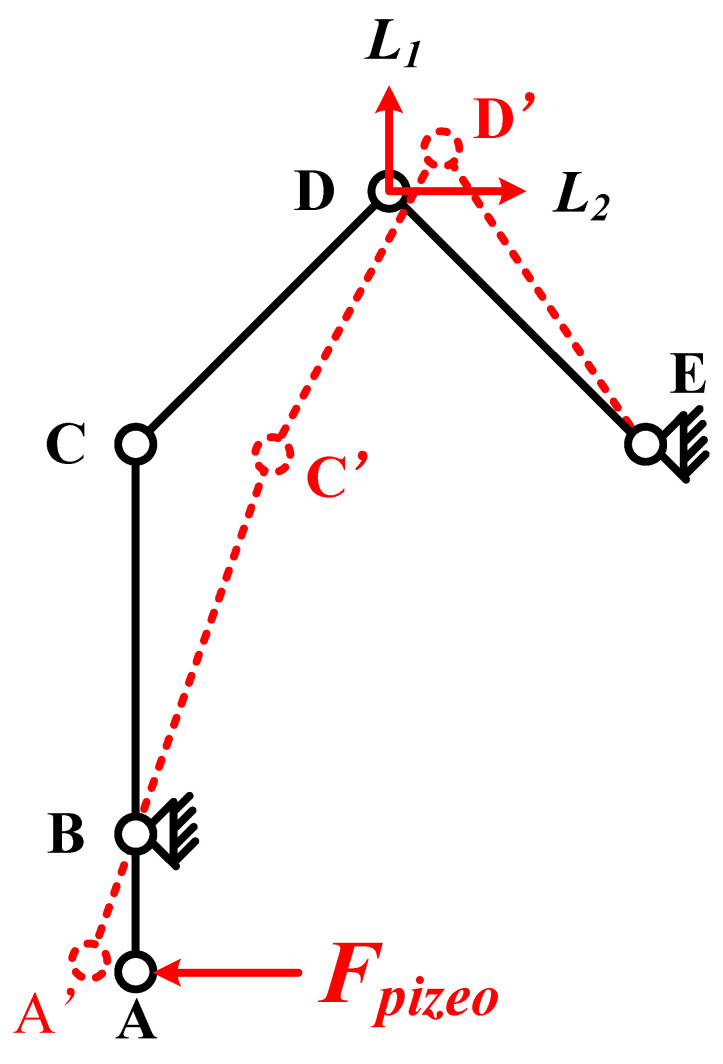
Kinematic model of the PEA.

In order to analyze the movement process of thePEA, a kinematic model is established, as shown in Figure 8. Point A is the contact stress point between the piezoelectric stack and the hinge structure. The contact stress point moves from point A to point A’. Point B is the fulcrum of the lever structure, corresponding to the hinge part with one end fixed on the bracket. Point C is the connection point between the lever displacement amplification structure and the triangular displacement amplification structure, which moves to point C’ in the driving process. Point D is the contact point with the mover in the driving foot and moves to point D’ during the driving process. E is the connection point between the triangular displacement amplification structure and the bracket, which is the fixed point.

Due to the action of the lever displacement amplification structure, the horizontal displacement from point C to C’ is significantly greater than that from point A to A’. The movement of point C drives the triangular displacement amplification structure to amplify the displacement and drives the contact point of the foot to move from D to D’, and the resulting displacement can be decomposed into L_1_ and L_2_, along with the vertical and horizontal directions. Through the above analysis, it can be seen that the tensile displacement generated by the piezoelectric stack produces the amplification effect of vertical and horizontal displacement at the contact point of the driving foot through the action of the lever displacement amplification structure and the triangular displacement amplification structure. The vertical displacement can be used as the clamping force to improve the friction force during the driving process. The horizontal displacement is used as the driving force of the mover.

#### 3.3.3. Force Analysis of the PEA

**Figure 9:**
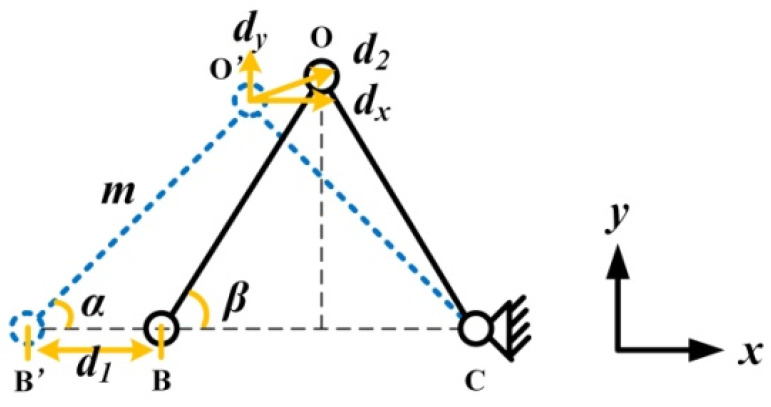
Simplified model of the triangular amplification structure.

In the structure of the PEA, the triangular amplification structure plays an important role, so it is analyzed separately. The simplified amplification triangular structure is shown in Figure 9. The circle in the figure represents each hinge, and the line represents the rigid connecting rod. Through the action of external force, the rotating flexible hinges in the magnifying mechanism change from the original state to the deformed state in the *x* and *y* directions.The displacement *d*_2_ generated by the driving foot is decomposed into *d_x_*> along the *x*-direction and *d_y_* along the *y*-direction.

According to the geometric relationship, the following equation can be obtained:

(11) $$m\sin\beta =m\sin\alpha +d_{y},$$

(12) $$m\cos\beta =m\cos\alpha -d_{x},$$

(13) $$2m\cos\alpha =2m\cos\beta +d_{1},$$

In the formula, m represents the length of the rigid connecting rod O’B’ (or OB). α is the angle between O’B’ and the x-direction. *β* is the angle between OB and the x-direction.The relationship between *d_x_* and *d*_1_ is calculated by the formula (12) and the formula (13):

(14) $$d_{1}=2d_{x},$$

According to the formula (14), the output displacement of the driving foot in the *x*-axis direction is only related to the input displacement, and the magnification in the *x*-direction is 0.5. The variable *β* can be eliminated by adding the squares of the formula (11) and the formula (12). The calculated *d_y_* is:

(15) $$d_{y}=-m\sin\alpha +\sqrt{m^{2}{\left(\sin\alpha \right)}^{2}+2d_{x}m\cos\alpha -d_{x}^{2}},$$

The displacement amplification factor *R* of the structure is expressed as:

(16) $$R=\frac{d_{y}}{d_{x}}=\frac{-2m\sin\alpha +\sqrt{4m^{2}{\left(\sin\alpha \right)}^{2}+4d_{1}m\cos\alpha -d_{1}^{2}}}{d_{1}},$$

It can be concluded from the formula (16), that the three parameters m, α and *d*_1_ determine the output characteristics of the triangular amplifying mechanism. m and α are the structural parameters of the triangular mechanism, which are invariant once designed and fabricated. So *d*_1_ is the only parameter that can be used to adjust the output displacement of the mover. In practice, since the input displacement *d*_1_ is very small compared to the structural parameters, the following simplified expression can be obtained after omitting the higher-order terms:

(17) $$d_{y}=d_{x}\cot\alpha ,$$

(18) $$R=\frac{d_{y}}{d_{x}}=\cot\alpha ,$$

A small value of *R* means that the output displacement is mainly used for driving, so a proper *R*-value can improve the driving efficiency. The displacement of the driving foot movement in this process is:

(19) $$d_{2}=\sqrt{d_{x}^{2}+d_{y}^{2}}=\frac{d_{1}}{2}\sqrt{1+\cot^{2}\alpha },$$

**Figure 10:**
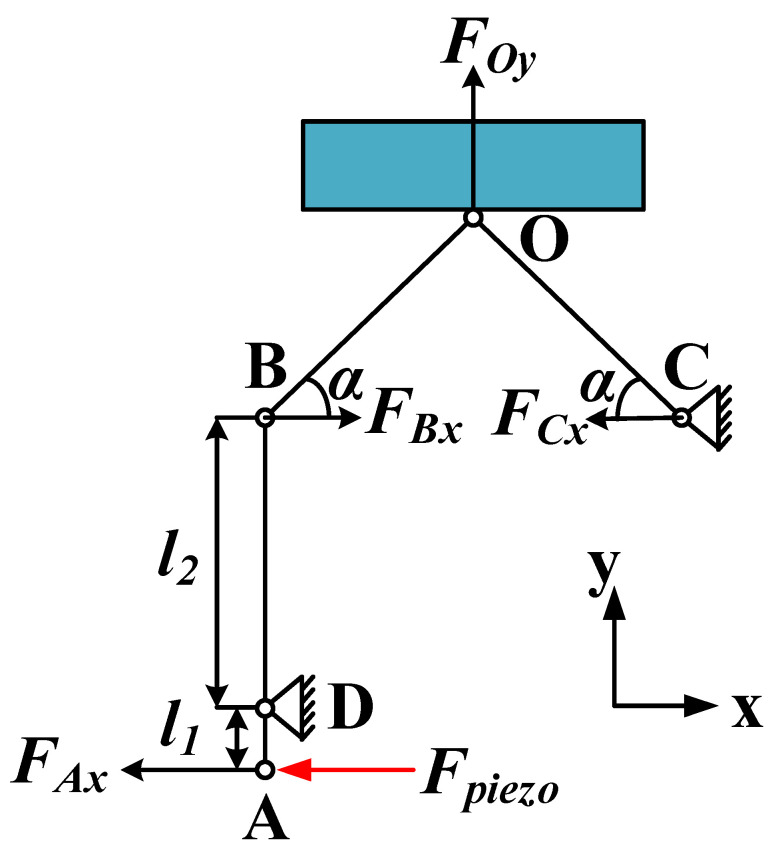
Force analysis of the PEA.

The force analysis of the PEA structure is carried out to determine that the two-stage displacement amplification structure can amplify the tensile displacement generated by the piezoelectric stack, as shown in Figure 10. The bar at the top of the figure represents the driven mover, and the lower part represents the simplified structural model of the PEA. Point O represents the contact point between the PEA and the mover. The *x*-axis represents the horizontal direction of the mover’s movement, and the *y*-axis represents the vertical direction of the applied clamping force.

The magnification ratio of the lever displacement amplification structure is [34]:

(20) $$\begin{array}{ccc} k & = & \frac{l_{2}}{l_{1}}, \end{array}$$

where *k* is the magnification ratio, *l*_1_ is the distance from point A to point D, and *l*_2_ is the distance from point D to point B.

The force analysis of the two-stage displacement amplification structure is as follows:

(21) $$\begin{array}{ccc} F_{.4x} & = & F_{pieeo}, \end{array}$$

(22) $$\begin{array}{ccc} F_{Bx} & = & \frac{F_{Ax}}{k}, \end{array}$$

(23) $$\begin{array}{ccc} F_{\mathrm{Cr}} & = & -F_{Bx}, \end{array}$$

(24) $$\begin{array}{c} F_{Oy}=F_{Bx}\tan\alpha -F_{Cx}\tan\alpha =\frac{2}{k}F_{pieeo\;}\tan\alpha , \end{array}$$

where *F_Ax_* is the force transmitted by the piezoelectric stack borne by the lever displacement amplification structure, and *F_piezo_* is the output force of the piezoelectric stack, ignoring the loss of force in this process. *F_Cx_* is the reaction force of the support on the triangular displacement amplification structure. According to the structural symmetry characteristics of the triangular displacement amplification structure, it is the same value and opposite direction as *F_Bx_. F_Oy_* is the clamping force applied by the driving foot on the mover. According to the formula, under the condition of *F_pizeo_* unchanged, the value of *F_Oy_* is related to k and α. The larger the value of k, the smaller the value of *F_Oy_.* The larger the value of α, the larger the value of *F_Oy_*. Since *F_Oy_* will not only affect the horizontal thrust of the actuator, but also easily jam the actuator and mover if the value is too large. Therefore, appropriate values of k and α should be selected to keep *F_Oy_* within a reasonable range. After choosing k as 2.1 and α as 60°, *F_Oy_* can be magnified by 1.6 times.

### 3.4. Simulation Analysis and Structure Optimization of the PEA

**Figure 11:**
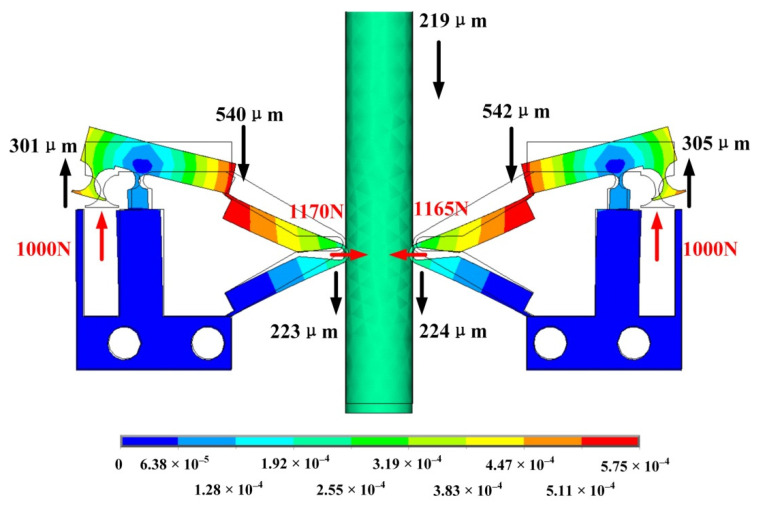
Deformation of the Amplification structure.

Through the static analysis of the simulation software, a 1000 N constant force is applied to the contact surface between the piezoelectric stack and the amplification structure, as shown in Figure 11. The magnification of the lever displacement amplification structure is 1.8 times, and the clamping forces generated between the driving feet and movers of the left and right PEAs are 1170 N and 1165 N, respectively, which are close to each other. The value of the clamping force is greater than the applied force, which shows that the structure plays a role in amplifying the clamping force. However, the magnification is less than 1.6 in theory.

In displacement amplification structures, displacement output and stress concentration are very important parameters. In order to produce a large output displacement, high-stress concentration will appear at the hinge of the amplifier. The stress concentration depends not only on the input of the amplifier but also on the hinge. As a lever mechanism with flexible hinges, once the radius of the corner hinge is reduced to zero, the highest flexibility can be obtained, but the stress concentration will increase sharply. Therefore, the hinge needs to be optimized.

**Figure 12:**
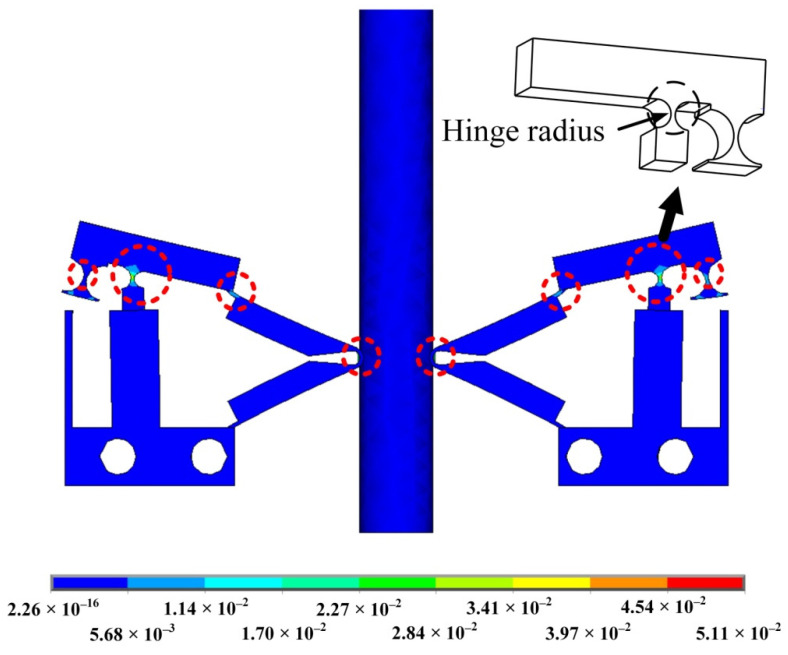
Stress distribution.

According to the stress distribution in Figure 12, the main stress generated by the actuator during operation is concentrated in the place surrounded by a red circle. The largest circle in the figure is the fulcrum of the lever amplification structure, where the stress is most obvious. Therefore, optimizing the hinge can improve the overall performance of the actuator. The following parametric analysis of the radius of the hinge (Figure 10) can obtainan ideal hinge radius parameter.

**Figure 13:**
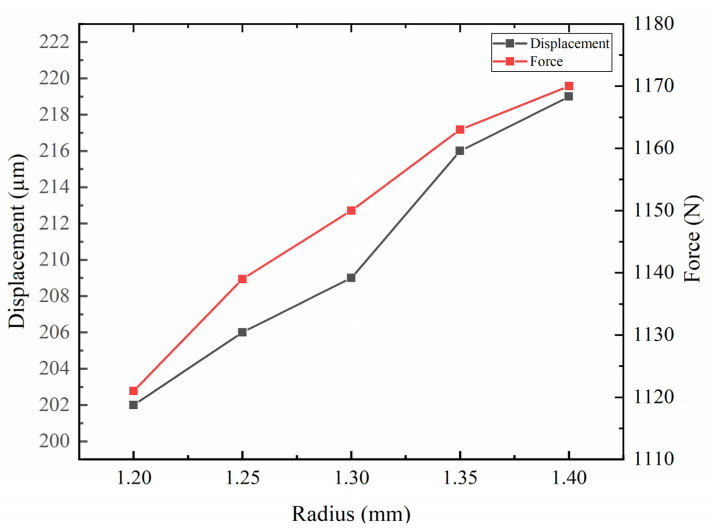
Parametric analysis of hinge radius.

Figure 13 is the parametric analysis diagram of the hinge radius. The left coordinate axis represents the displacement of the mover. The right axis represents the clamping force between the driving foot and the mover. The lower axis represents the radius of the hinge. According to the curve, it can be seen that with the increase in the hinge radius, the displacement of the actuator increases, and the clamping force between the driving foot and the actuator also increases. A hinge radius of 1.4 mm is selected.

### 3.5. Operation Principle of the PEA

**Figure 14:**
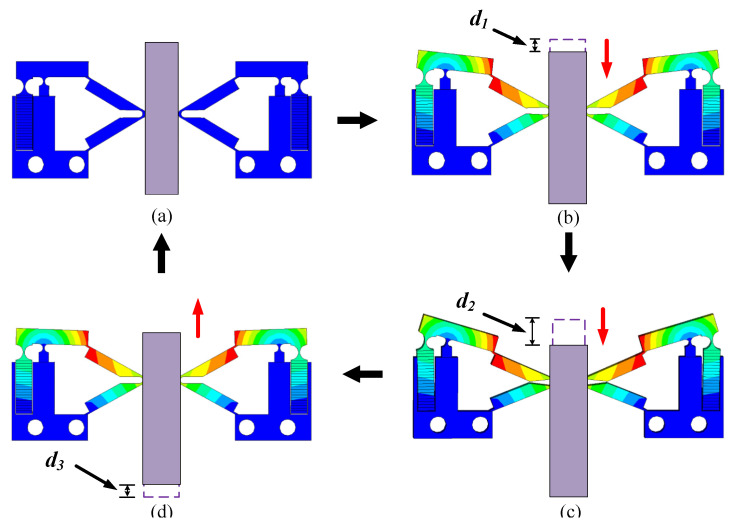
Operation process of the PEAs in one cycle; (**a**) Initial state; (**b**) Steady state; (**c**) Maximum steady state; (**d**) Sliding state.

The operation process of the PEAs in one cycle is shown in Figure 14. Figure 14a shows the initial state of the actuator without voltage. Figure 14b shows a certain amount of deformation that the actuator begins to produce after the voltage is first applied to the actuator. The driving foot drives the mover to move downward, resulting in a displacement of *d*_1_. With the increase in voltage, the deformation of the actuator reaches the maximum. At this time, the displacement produced by the driving foot to the mover reaches the maximum value in this cycle. The mover moves down the displacement of d_2_ on the basis of *d*_1_, as shown in Figure 14c. Finally, the driving voltage is gradually reduced to zero. The actuator is restored to its initial state, as shown in Figure 14d. In this process, due to there still being a certain clamping force between the driving foot and the mover, the mover will produce a small amount of reverse displacement d_3_ under the action of friction. To sum up, the displacement generated by the actuator in one cycle is $\begin{array}{ccc} \Delta d & = & d_{1}+d_{2}-d_{3} \end{array}$. Under the action of two actuators, the mover presents a stepping motion in multiple cycles.

**Figure 15:**
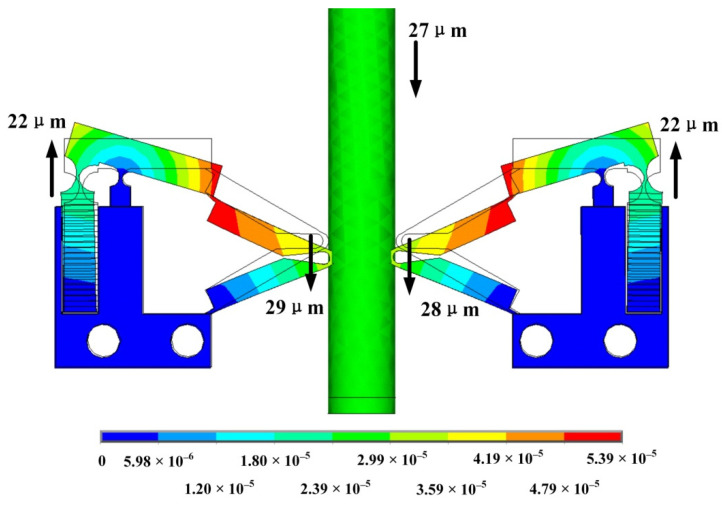
ThePEA deformation under maximum driving.

According to the characteristics of the piezoelectric stack, the maximum voltage that can be applied to the PEA is 150 V. In order to calculate the distance that the mover can be driven under the maximum voltage, it is necessary to conduct a static analysis of the PEA to which the voltage is applied, as shown in Figure 15. It can be obtained that the maximum displacement of the mover driven by the PEA will not exceed 27 mm in one cycle. Because the driving mode of the PEA is longitudinal vibration, it can work under voltages of different frequencies. Therefore, selecting the driving voltage at a low frequency can improve the resolution of the actuator. With the reasonable adjustment of the voltage amplitude V_p-p_, the displacement of the mover can be changed more accurately.

## 4. Electromagnetic Drive Part

### 4.1. Structure of the EMA

**Figure 16:**
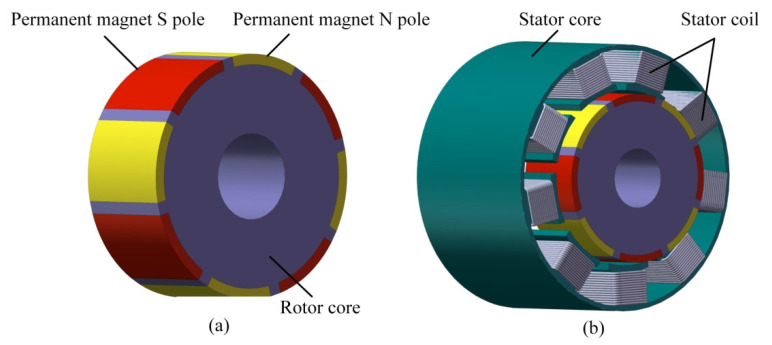
Structure of the EMA; (**a**) Rotor model; (**b**) Stator model.

The fractional slot concentrated winding permanent magnet actuator has the advantages of excellent torque characteristics, small positioning torque, and small torque fluctuation. As shown in Figure 16, the EMA designed in this paper has nineslots, four pole pairs, eight permanent magnets that adopt surface patch type, the magnetic field direction of the magnetic pole is radial, the stator core is slotted to form teeth, each tooth end is provided with pole shoes, the coil is directly wound on the stator tooth stage, and the pitch of all coils is 1, which is called concentrated winding.

**Figure 17:**
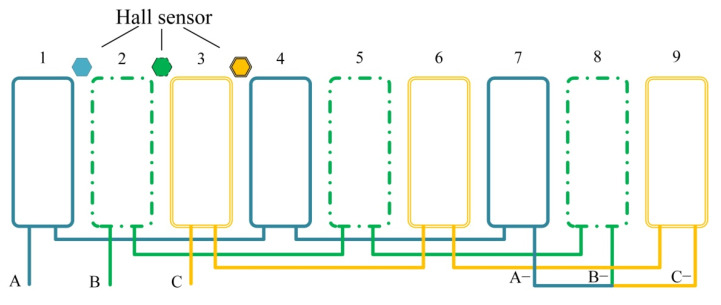
Coil winding mode.

The coil end length of the concentrated winding is short, the copper loss is small, the efficiency is high, the winding has no overlap, the phase-to-phase insulation is good, the coil is easy to be mechanically offline, and the production cost is reduced. The coil winding mode is shown in Figure 17. Three blue coils are connected in series to form A-phase winding, three green coils are connected in series to form B-phase winding, and three yellow coils are connected in series to form C-phase winding. Nine coils form a three-phase winding, and the ends of the three phases are connected to form a star connection method.

**Figure 18:**
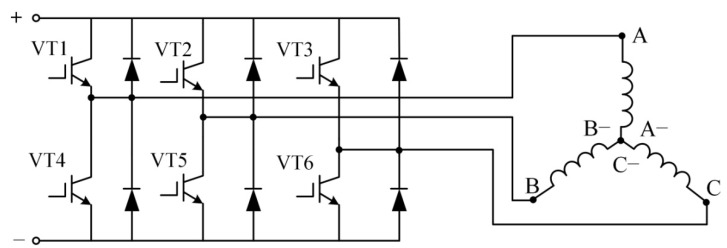
Drive circuit.

The driving power supply of the actuator is composed of a three-phase bridge circuit, as shown in Figure 18. Unlike the three-phase reverse step actuator or three-phase synchronous actuator, the input of the EMA is not a sine wave, and only two are connected at each time. The switching of the switching transistor is controlled by the position detection device, and the hall element is used to detect the polarity and turnover of the permanent magnet. The three Hall elements are, respectively installed at the three leading positions of the three-phase coil, as shown in Figure 17. The hall element is installed at the gap between the two tooth poles of the stator. When the junction of the two magnetic poles of the rotor passes through the Hallsensors, the Hall sensors detect the polarity change and send a signal to control the driving circuit to switch the three-phase current. The magnetic field generated by the two-phase coil attracts the rotor to rotate.

### 4.2. Analysis of Electromagnetic Drive

The EMA consists of an electromagnetic stator and an electromagnetic rotor, as shown in Figure 16. The electromagnetic stator is composed of stator windings and an iron core. The electromagnetic rotor is composed of permanent magnets and an iron core. The material of permanent magnets is Nd-Fe-B-35, and the magnetization mode of permanent magnets is transverse magnetization. The permanent magnet is embedded in the rotor core. The rotor core is integrated with the transmission rod. One end of the transmission rod is used as an output shaft, and the other end is connected with the bearing device so that the rotation of the rotor is not affected.

Since the EMA and PEA are assembled as a whole, the size ratio of the two should match. The volume of PEA is relatively small. Therefore, when selecting theEMA, it should not only have the characteristics of high-power density, high efficiency, and a high power factor, but also realize the energy-saving, miniaturization, and lightweight characteristics of the actuator.

Concerning the choice of stator slot number and extreme logarithm, the actuator needs to meet the characteristics of low speed and large torque. The formula for the number of slots per pole and phase of the actuator is:

(25) $$\begin{array}{ccc} q & = & \frac{Z}{2pm}, \end{array}$$

The number of phases of the actuator is m, the total number of slots of the stator is *Z*, and the number of pole pairs of the permanent magnet rotor is *p*. The actuator structure is distributed winding at q > 1 and concentrated winding at q ≤ 1. When q is an integer, it is an integer slot winding, and when q is a fraction, it is a fractional slot winding.

The permanent magnet synchronous actuator with a high pole number has the characteristics of small pole distance, high slot full rate, simple wire embedding process, and small torque fluctuation. Therefore, the selected number of the pole is higher; that is, the p-value is larger. The stator of a low-speed permanent magnet synchronous actuator mostly adopts fractional slot concentrated winding, and Q is less than or equal to 1 and is fractional. The stator adopts centralized winding, which has the characteristics of simple structure, short winding end length, and high energy density. The fractional slot can increase the frequency of the fundamental wave of the cogging torque and significantly reduce the pulsing momentum of the cogging torque, so as to reduce the vibration and noise caused by the cogging torque to the actuator. However, the distribution of windings at all levels is asymmetric, so the effective torque component of the actuator is partially offset, and the average torque of the actuator will be reduced accordingly.

Because the odd slot actuator has the characteristics of high torque density and low torque ripple, in order to compensate for the reduction in the average torque of the fractional slot concentrated winding, the number of slots is selected as odd. This not only cooperates with the low speed and high torque characteristics of PEA, but also reduces the noise and impact on parts caused by vibration and improves the overall life durability. Therefore, it can be better combined with the piezoelectric part.

In the selection of stator slot number *Z* and pole pair P, the formula is as follows:

(26) $$\begin{array}{ccc} Z & = & 2p\pm 1, \end{array}$$

After comprehensive consideration, when the combination of *Z* = 9, *p* = 4 or 5 is selected, the harmonic winding coefficient is the largest. Due to the amplitude of magnetomotive force being inversely proportional to its polar logarithm, the amplitude of *p* = 4 is relatively large. When *p* = 4, the 4-pole magnetomotive force is the fundamental wave, and the 5-pole magnetomotive force is the influential harmonic. It is the opposite when *p* = 5, so it is more appropriate to choose *p* = 4.

The amplitude of the magnetomotive force at 4-pole is:

(27) $$F_{mc4}=\frac{2\sqrt{2}IN}{\pi }\left[\int_{0}^{\frac{\pi }{9}}\frac{8}{9}\cos4\alpha d\alpha -\int_{\frac{\pi }{9}}^{\pi }\frac{1}{9}\cos4\alpha d\alpha \right]=\frac{\sqrt{2}IN}{2\pi }\sin\frac{4}{9}\pi ,$$

where *I* is the current flowing through the coil and *N* is the number of coil turns. The magnetomotive force generated by a single coil is:

(28) $$\begin{array}{ccc} F_{c}\left(t,\alpha \right) & = & \sin\omega t*F_{mc4}*\cos4\alpha , \end{array}$$

where *ω* is the angular frequency. When *z* = 9, three coils of the same phase are alternately connected in series according to positive and negative to form a coil group. The distribution coefficient of harmonic winding can be obtained by adding each vector:

(29) $$\begin{array}{ccc} k_{q4} & = & \frac{1}{3}\left(2\cos4\alpha_{0}-1\right) \end{array}$$

The double-layer winding is adopted, the mechanical angle of the spatial difference between the coil axes of the two windings on the adjacent teeth is $\begin{array}{ccc} \alpha_{0} & = & \frac{2\pi }{9} \end{array}$, and the harmonic magnetomotive force amplitude of the coil group is:

(30) $$\begin{array}{c} F_{mq4}=3F_{mc}k_{q_{4}}=\frac{\sqrt{2}IN}{2\pi }\sin\frac{4}{9}\pi *\left(2\cos\frac{8\pi }{9}-1\right), \end{array}$$

For odd slot actuators, there is only one coil group per phase, so the harmonic magnetomotive force of the coil group is the harmonic magnetomotive force of the phase winding.

(31) $$\begin{array}{ccc} F_{mq4} & = & F_{m\varphi 4,} \end{array}$$

when the symmetrical three-phase winding is connected with the symmetrical three-phase alternating current (AC) with an angle of 2π/3 different from each other in time, the magnetomotive force generated by the three-phase winding is:

(32) $$\begin{array}{c} \left\{\begin{array}{c} F_{A4}\left(t,\alpha \right)=F_{m\varphi 4}\cos4\alpha \sin\omega t\\ F_{B4}\left(t,\alpha \right)=F_{m\varphi 4}\cos4\left(\alpha -\frac{2\pi }{3}\right)\sin\left(\omega t-\frac{2\pi }{3}\right)\\ F_{C4}\left(t,\alpha \right)=F_{m\varphi 4}\cos4\left(\alpha -\frac{4\pi }{3}\right)\sin\left(\omega t-\frac{4\pi }{3}\right), \end{array}\right. \end{array}$$

The magnetomotive force of the harmonic synthesis of three-phase winding is:

(33) $$\begin{array}{ccc} F_{4}\left(t,\alpha \right) & = & F_{A4}\left(t,\alpha \right)+F_{B4}\left(t,\alpha \right)+F_{C4}\left(t,\alpha \right), \end{array}$$

(34) $$\begin{array}{ccc} F_{4}\left(t,\alpha \right) & = & \frac{3}{2}F_{m\varphi 4}\sin\left(\omega t-4\alpha \right), \end{array}$$

The amplitude is 3/2-times the amplitude of a single-phase winding magnetomotive force. The rotating magnetomotive force moves in the positive direction of α. The mechanical angular velocity is *ω*/4.

### 4.3. Magnetic Field Analysis in the Empty Air Gap Region

**Figure 19:**
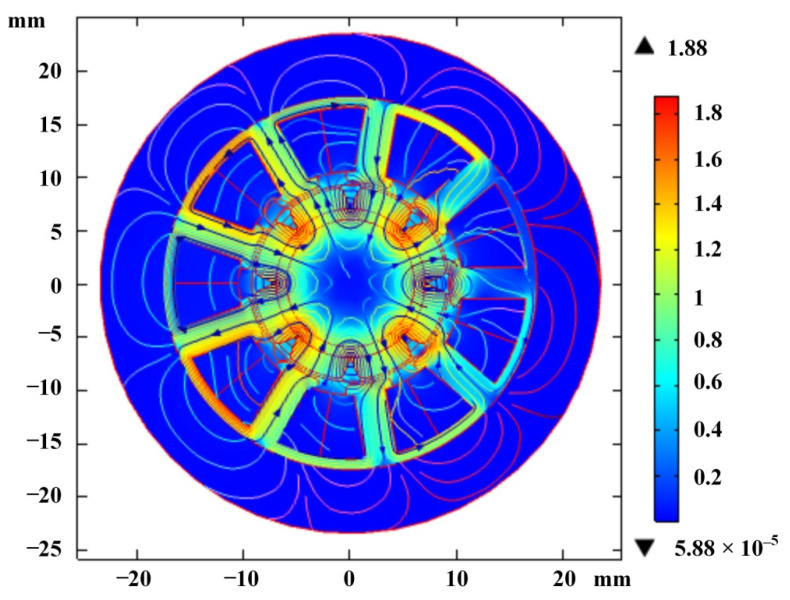
Distribution of magnetic flux density.

The magnetic flux density distribution under the static state is shown in Figure 19. The magnetic field is concentrated in the permanent magnet and air gap, so the magnetic fields in the permanent magnet and air gap are analyzed, respectively.

Assuming that the permeability of the stator and rotor core is infinite, the magnetic field in the permanent magnet area can be expressed by the Poisson equation:

(35) $$\begin{array}{ccc} \frac{\partial^{2}A_{z1}}{\partial r^{2}}+\frac{1}{r}\frac{\partial A_{z1}}{\partial r}+\frac{1}{r^{2}}\frac{\partial^{2}A_{z1}}{\partial \theta^{2}} & = & \frac{-\mu }{r}\left(M_{\theta }-\frac{\partial M_{r}}{\partial \theta }\right), \end{array}$$

The general solution is obtained after solving:

(36) $$A_{z1}=\sum_{k}\left[A_{1}{\left(\frac{r}{R_{m}}\right)}^{k}+B_{1}{\left(\frac{r}{R_{r}}\right)}^{-k}\right]\cos(k\theta )+\sum_{k}\left[C_{1}{\left(\frac{r}{R_{m}}\right)}^{k}+D_{1}{\left(\frac{r}{R_{r}}\right)}^{-k}\right]\sin(k\theta )+A_{p},$$

In the above formula, *r* and *θ* togetherrepresent the position information of any point in the permanent magnet area, where *r* is the radial length and *θ* is the circumferential angle. *M_r_* and *M_θ_* are the radial component and the tangential component of the magnetization, respectively. The radial component of the magnetic density in the permanent magnet region is:

(37) $$\begin{array}{l} B_{1r}=\frac{1}{r}\frac{\partial A_{z1}}{\partial \theta }\\ =-\frac{1}{r}\sum_{k}k(C_{1k}A_{1}+C_{2k}M_{ack}-C_{3k}M_{rsk})\sin(k\theta )+\frac{1}{r}\sum_{k}k(C_{1k}C_{1}+C_{2k}M_{ask}+C_{3k}M_{rsk})\cos(k\theta ), \end{array}$$

The Laplace equation of the air gap region in the polar system is:

(38) $$\frac{\partial^{2}V}{\partial r^{2}}+\frac{1}{r}\frac{\partial V}{\partial r}+\frac{1}{r^{2}}\frac{\partial^{2}V}{\partial \theta^{2}}=0,$$

The function is solved by the method of separating variables. The general solution of scalar magnetic potential is:

(39) $$V=\sum_{k}^{}\left[A_{2}{\left(r/R_{s}\right)}^{k}+B_{2}{\left(r/R_{m}\right)}^{-k}\right]\cos\left(k\theta \right)+\sum_{k}^{}\left[C_{2}{\left(r/R_{s}\right)}^{k}+D_{2}{\left(r/R_{m}\right)}^{-k}\right]\sin\left(k\theta \right),$$

*R_S_* is the radius from the center to the inner frame of the stator, *R_m_* is the radius from the center to the outer frame of the permanent magnet, and *A*_2_, *B*_2_, *C*_2,_ and *D*_2_ are the coefficients to be calculated. The radial component of the magnetic density in the air gap region is:

(40) $$B_{2r}=-\sum_{k}k\left[\frac{A_{2}}{R_{s}}{\left(\frac{r}{R_{s}}\right)}^{k-1}+\frac{B_{2}}{R_{m}}{\left(\frac{r}{R_{m}}\right)}^{-k-1}\right]\sin(k\theta )+\sum_{k}k\left[\frac{C_{2}}{R_{s}}{\left(\frac{r}{R_{s}}\right)}^{k-1}+\frac{D_{2}}{R_{m}}{\left(\frac{r}{R_{m}}\right)}^{-k-1}\right]\cos(k\theta ),$$

The radius *R_r_* of the rotor yoke, the radius *R_m_* of the eccentric permanent magnet, and the radius *R_s_* from the rotor center to the inner edge of the stator can be optimized according to the above formula.

**Figure 20:**
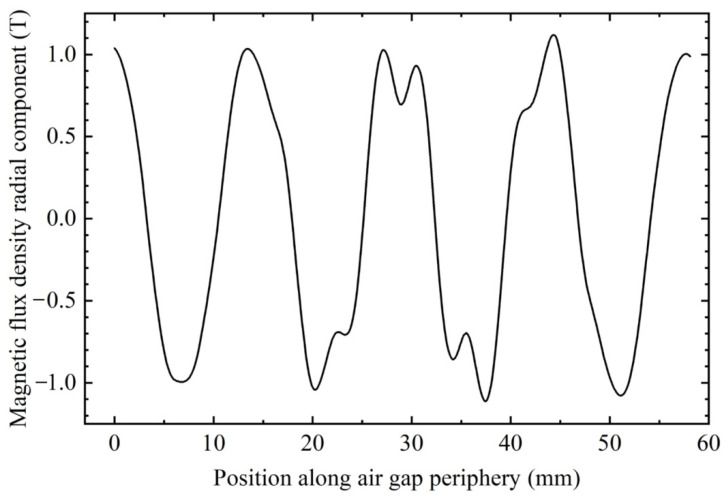
Relationship between the radial component of air-gap flux and the peripheral position of airgap.

The spatial distribution of air gap flux in a synchronous actuator is very important. Figure 20 shows the variation curve of radial flux along the periphery of theair gap. It can be seen from the figure that the magnetic flux presents an approximate sinusoidal distribution, which shows that the actuator can avoid the introduction of higher harmonics.

**Figure 21:**
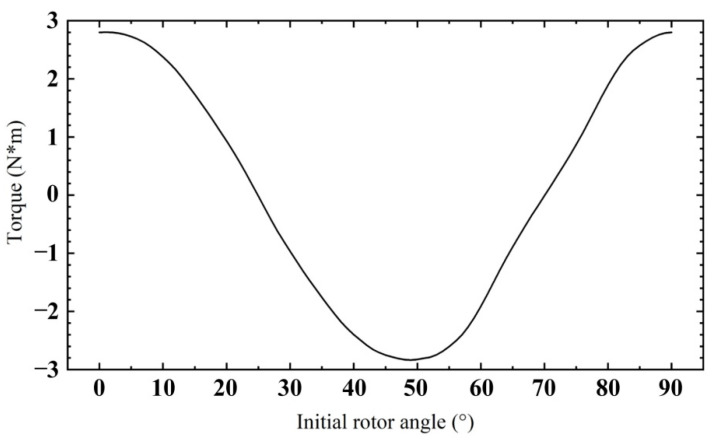
Relationship between rotor torque and initial rotor angle.

The torque of the rotor will change according to the relative position between the stator and the rotor, so adjusting the appropriate rotor angle can provide the maximum torque output for the rotor. According to the relationship curve between rotor torque and rotor initial angle in Figure 21, it can be concluded that strong torque can be generated when the initial angle is 49°. The generated torque is 2.9 N·m.

## 5. Experimental Analysis

**Figure 22:**
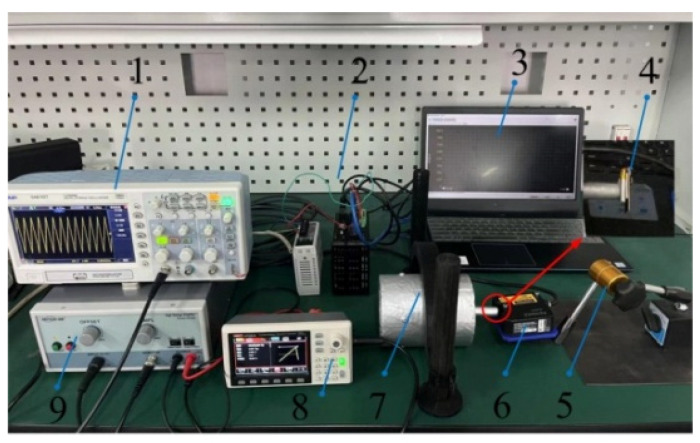
The experimental platform (1. Oscilloscope, 2. Controller, 3. PC, 4. Attitude Sensor 5. Magnetic Base, 6. Laser Sensor, 7. Prototype, 8. Signal Generator, 9. Voltage Amplifier).

The experimental platform is composed of a signal generator, a voltage amplifier, a laser sensor, an oscilloscope, an attitude sensor, a personal computer (PC), and the proposed hybrid drive actuator, as shown in Figure 22. The function of the signal generator is to provide a power supply with an appropriate waveform for PEAs. The voltage amplifier is connected with the signal generator to adjust the voltage V_p-p_ and apply the adjusted voltage to the piezoelectric stack of PEAs. The laser sensor is used to measure the displacement, velocity, and acceleration of the transmission rod. The laser sensor is fixed on the magnetic base used to adjust the position of the sensor. The attitude sensor is used to detect the rotation speed of the rod. The PC is connected to the sensors to extract the data detected by the sensors.

According to the data transmitted from the attitude sensor to the PC, when a 10 A current is applied to the EMA, the rotor can produce a maximum speed of 9 rad/s.

**Figure 23:**
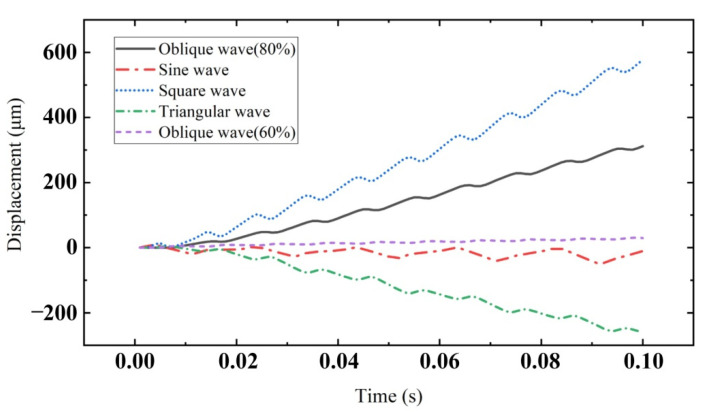
Displacement of mover under different voltage signal waveforms.

Figure 23 shows the relationship between the displacement of the rod and time under different voltage signal waveforms. Through the comparison in the figure, it can be concluded that under the action of the same time and excitation V_p-p_, the displacement generated by the square wave is the largest. Therefore, when the actuator needs to output a large displacement within a certain time, the square wave can be selected for output. The triangular wave is an oblique wave with 50% symmetry. In the figure, the triangular wave produces the maximum reverse displacement. The oblique wave with 80% symmetry produces large forward displacement. Therefore, the actuator can be driven forward and backward according to the change in oblique wave symmetry to make better use of the actuator.

**Figure 24:**
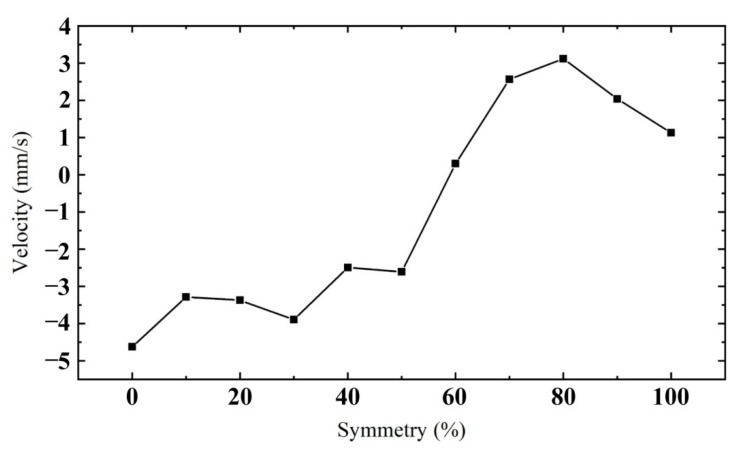
Relationship between mover velocity and oblique wave symmetry.

Figure 24 shows the relationship between the velocity of the mover and the symmetry of the oblique wave. The excitation amplitude applied to PEAs is 100 V. It can be seen from the figure that when the symmetry is 0%, the reverse velocity generated by the mover obtains its maximum. When the symmetry is 80%, the forward velocity of the mover obtains its maximum. According to changing the symmetry of oblique wave excitation, the characteristic of the driving direction of the actuator to the mover can be changed, which is convenient for the later control work. The symmetry of oblique wave excitation is selected as 80%, and the forward motion of the mover is analyzed.

**Figure 25:**
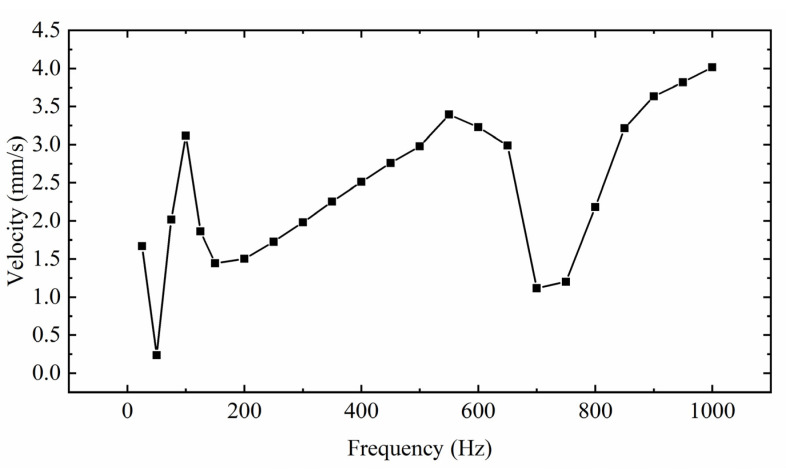
Relationship between the velocity of mover and excitation frequency.

When the symmetry of oblique wave excitation is 80% and V_p-p_ is 100 V, the relationship between mover velocity and excitation frequency is shown in Figure 25. It can be seen from the figure that there are three peaks in the curve during the change process, which are 100 Hz, 550 Hz, and 1000 Hz, respectively. The fluctuation before and after 550 Hz is relatively gentle, and the mover speed is relatively high. The speed is higher at 1000 Hz, but the higher the frequency, the higher the accuracy of control. It mainly studies the output performance at low frequency, so 100 Hz is selected for the following analysis.

**Figure 26:**
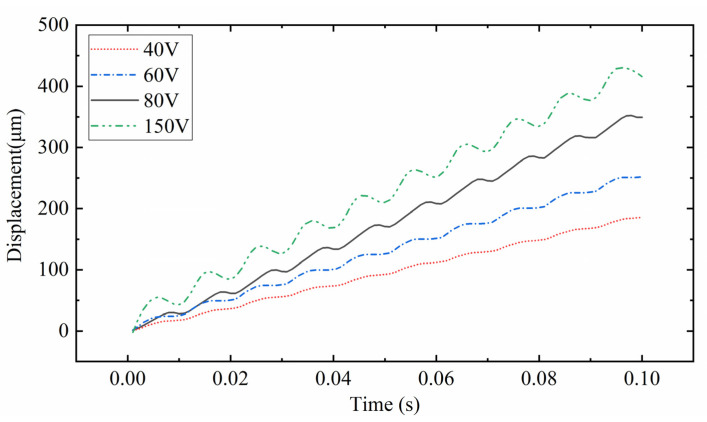
Relationship between displacement and time of mover under different excitation V_p-p_.

Figure 26 shows the relationship between the displacement and time of the mover under different V_p-p_. The frequency of voltage excitation is 100 Hz. As shown in the figure, each displacement track is a stepped rising track. The curve from top to bottom in the figure is the curve when the excitation V_p-p_ is 150 V, 80 V, 60 V, and 40 V. The greater the voltage excitation V_p-p_, the more obvious the reverse displacement produced by the mover. The reverse displacement hinders the movement of the mover and affects the stability of PEAs. Therefore, when there are no requirements for speed and displacement, the smaller V_p-p_ can be selected as far as possible to ensure the smooth movement of the mover.

**Figure 27:**
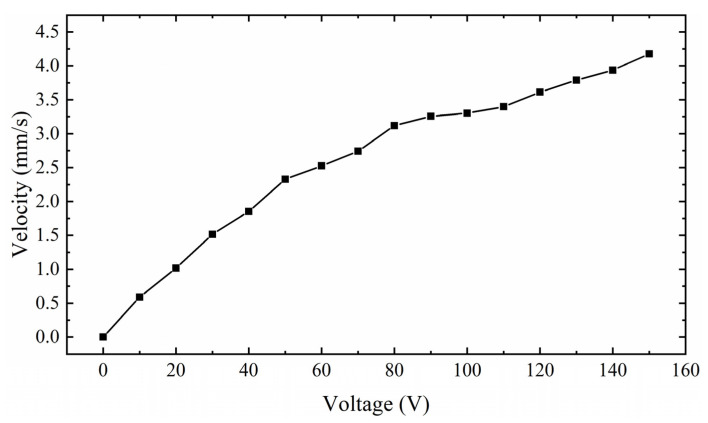
Relationship between mover speed and voltage excitation V_p-p_.

Figure 27 shows the relationship between the velocity of the mover and the excitation amplitude. With the increase in excitation amplitude, the velocity of the mover increases gradually, and its increasing trend increases rapidly at first and then gently. On this basis, it is found that when V_p-p_ is about 100 V, the velocity of the mover is relatively stable, and it is the initial part of the slow velocity growth region. Therefore, to consider the working efficiency and stability of the actuator, 100 V is selected as V_p-p_ excited by the PEAs. It can output a relatively stable speed of 3.4 mm/s. It is proved that the actuator has good output performance.

**Figure 28:**
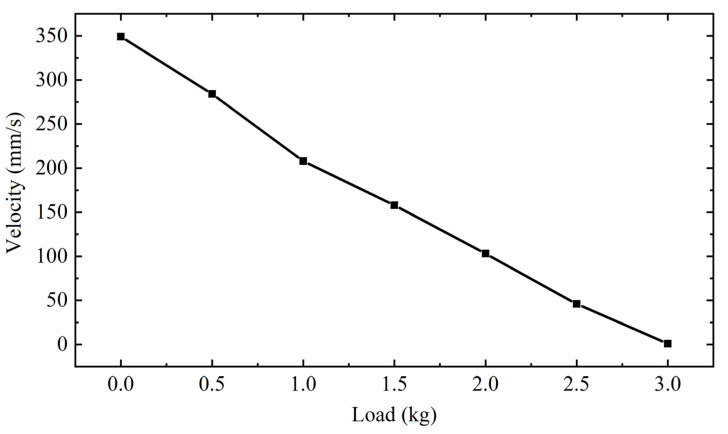
Relationship between mover speed and load capacity.

Figure 28 shows the relationship between the speed of the mover and the load capacity of the actuator. The applied voltage excitation is 150 V, 3.4 kHz. At this time, the actuator works in the high-frequency region of excitation, and the excitation amplitude is the largest, so the driving speed is the fastest. It can be seen from the figure that under no load, the speed of the mover is 348 mm/s. With the increase in load weight, the speed of the mover decreases gradually. Finally, when the load is 3 kg, the speed of the mover is close to 0. Therefore, the load capacity of the actuator is 3 kg. It is proved that the actuator has a high load capacity.

## 6. Conclusions

This paper presents a hybrid drive actuator. Because the actuator adopts an electromagnetic–piezoelectric hybrid actuator, it combines the characteristics of PEA and EMA with small volume, strong load capacity, strong anti-interference ability, and high displacement resolution. Therefore, in the corresponding workplace, it can replace the traditional single drive actuator. In addition, the driving principle of the actuator is analyzed, and the running state of the actuator is described. The structure is optimized and analyzed to improve the performance of the actuator. Finally, the driving performance of the actuator is tested experimentally. The experimental results show that the actuator can output a stable speed of 3.4 mm/s at low frequency, the maximum driving speed can reach 348 mm/s, the load capacity is 3 kg, and the maximum torque is 2.9 N·m and the maximum speed is 9 rad/s. It proves the rationality of the combination of a piezoelectric drive and electromagnetic drive and provides a design idea for the design of a piezoelectric–electromagnetic hybrid drive actuator in the future.

To sum up, the rationality and feasibility of the actuator are verified by simulation and experimental methods, and the idea of a multi-DOF ultrasonic actuator is extended. It is worth noting that the actuator is driven independently by an electromagnetic–piezoelectric actuator, and the two driving parts independently drive the transmission rod. The next work will work towards the direction of common electromagnetic–piezoelectric-driven actuators. In addition, vibration also has a certain impact on the actuator, which will be further developed in future work.

## Data Availability

All data included in this study are available upon request by contact with the corresponding author.
